# Coculture engineering for efficient production of vanillyl alcohol in *Escherichia coli*

**DOI:** 10.1007/s42994-022-00079-0

**Published:** 2022-09-05

**Authors:** Meichen Yang, Hao Meng, Xianglai Li, Jia Wang, Xiaolin Shen, Xinxiao Sun, Qipeng Yuan

**Affiliations:** State Key Laboratory of Chemical Resource Engineering, Beijing University of Chemical Technology, Beijing, 100029 China

**Keywords:** Vanillyl alcohol, Coculture engineering, Modular optimization, Methylation, S-adenosylmethionine

## Abstract

Vanillyl alcohol is a precursor of vanillin, which is one of the most widely used flavor compounds. Currently, vanillyl alcohol biosynthesis still encounters the problem of low efficiency. In this study, coculture engineering was adopted to improve production efficiency of vanillyl alcohol in *E. coli*. First, two pathways were compared for biosynthesis of the immediate precursor 3, 4-dihydroxybenzyl alcohol in monocultures, and the 3-dehydroshikimate-derived pathway showed higher efficiency than the 4-hydroxybenzoate-derived pathway. To enhance the efficiency of the last methylation step, two strategies were used, and strengthening S-adenosylmethionine (SAM) regeneration showed positive effect while strengthening SAM biosynthesis showed negative effect. Then, the optimized pathway was assembled in a single cell. However, the biosynthetic efficiency was still low, and was not significantly improved by modular optimization of pathway genes. Thus, coculturing engineering strategy was adopted. At the optimal inoculation ratio, the titer reached 328.9 mg/L. Further, gene *aroE* was knocked out to reduce cell growth and improve 3,4-DHBA biosynthesis of the upstream strain. As a result, the titer was improved to 559.4 mg/L in shake flasks and to 3.89 g/L in fed-batch fermentation. These are the highest reported titers of vanillyl alcohol so far. This work provides an effective strategy for sustainable production of vanillyl alcohol.

## Introduction

Vanillin is one of the most important aromatic flavor compounds widely used in foods, beverages, perfumes, cosmetics and pharmaceuticals. Its annual global market reaches twenty thousand tons. Natural vanillin is extracted from Vanilla pods. The price is between $1200 and 4000 per kilogram, and the output can fulfill less than 1% of the market demand. Currently, vanillin is mainly produced by chemical synthesis from guaiacol and lignin, and the average price is less than $15 per kilogram (Banerjee and Chattopadhyay 2019; Martău et al. 2021). However, this method has the problem of environment pollution. The increasing health- and nutrition-consciousness of the customers has led to a growing interest to produce natural vanillin by biotechnology-based approaches.

Bioconversion studies have been conducted for vanillin production using ferulic acid, eugenol and isoeugenol as the major substrates (Kaur and Chakraborty 2013; Ma et al. 2022; Yamada et al. 2008), among which the ferulic acid-based route shows the highest efficiency. For example, a vanillin-tolerant bacterium *Amycolatopsis* sp. ATCC 39,116 was engineered by disrupting the catabolism gene *vdh* and enhancing the anabolism genes *ech* and *fcs*, leading to the production of 19.3 g/L of vanillin with a molar yield of 94.9% from ferulic acid (Fleige et al. 2016). However, the price for ferulic acid is relatively high (approximately $100 per kilogram), and thus only 500–600 tons of vanillin is produced via bioconversion annually for the high-end market.

Besides bioconversion, biosynthesis of vanillin or its related compounds (vanillate and vanillyl alcohol) from cheap carbon sources such as glucose has also been investigated. In 1998, a vanillate biosynthetic pathway was constructed in *E. coli* by heterologous expression of a 3-dehydroshikimate (3-DHS) dehydratase and a catechol-O-methyltransferase. Vanillate produced was further converted to vanillin by a carboxylic acid reductase (CAR) in vitro (Li and Frost 1998). De novo production of vanillin was first achieved in both *S. cerevisiae* and *Schizosaccharomyces pombe* by co-expressing three similar enzymes as above, producing 65 and 45 mg/L of vanillin from glucose, respectively (Hansen et al. 2009). *E. coli* contains many endogenous enzymes with aldehyde reductase activity, which would lead to formation of vanillyl alcohol instead of vanillin in vivo. To solve this problem, a recombinant *E. coli* with reduced aromatic aldehyde reduction (RARE) was engineered by inactivating three aldo–keto reductases and three alcohol dehydrogenases, leading to a 55-fold increase in vanillin titer to 119 mg/L (Kunjapur et al. 2014). In addition, the reaction catalyzed by O-methyltransferase was verified to be rating–limiting, and was strengthened by either enhancing supply or regeneration of the methy doner S-adenosylmethionine (SAM). As a result, the titer of vanillate reached 272 mg/L in shake flask experiment. Previously, our group designed a novel vanillyl alcohol biosynthetic pathway, which consists of three heterologous enzymes 4-hydroxybenzoate (4-HBA) hydroxylase (PobA), CAR and caffeate O-methyltransferase (COMT). The engineered *E. coli* with this pathway produced 240.69 mg/L of vanillyl alcohol with the accumulation of 282.53 mg/L of 3, 4-dihydroxybenzyl alcohol (3,4-DHBA) (Chen et al. 2017). As indicated above, the biosynthetic efficiency of vanillin/vanillate/vanillyl alcohol still requires significant improvement for practical application.

When an entire pathway is accommodated in one cell, it may cause heavy metabolic burden, and balancing the gene expression and the cofactor/co-substrate supply is laborious and sometimes difficult. Thus, the modular coculture engineering has been developed, which divides the full pathway into modules and distribute them into two or more cells. The strategy has been applied to the production of various chemicals such as apigenin, rosmarinic acid, salidroside and phenol (Thuan et al. 2018; Li et al. 2019b, 2022; Liu et al. 2018; Guo et al. 2019).

**Fig. 1:**
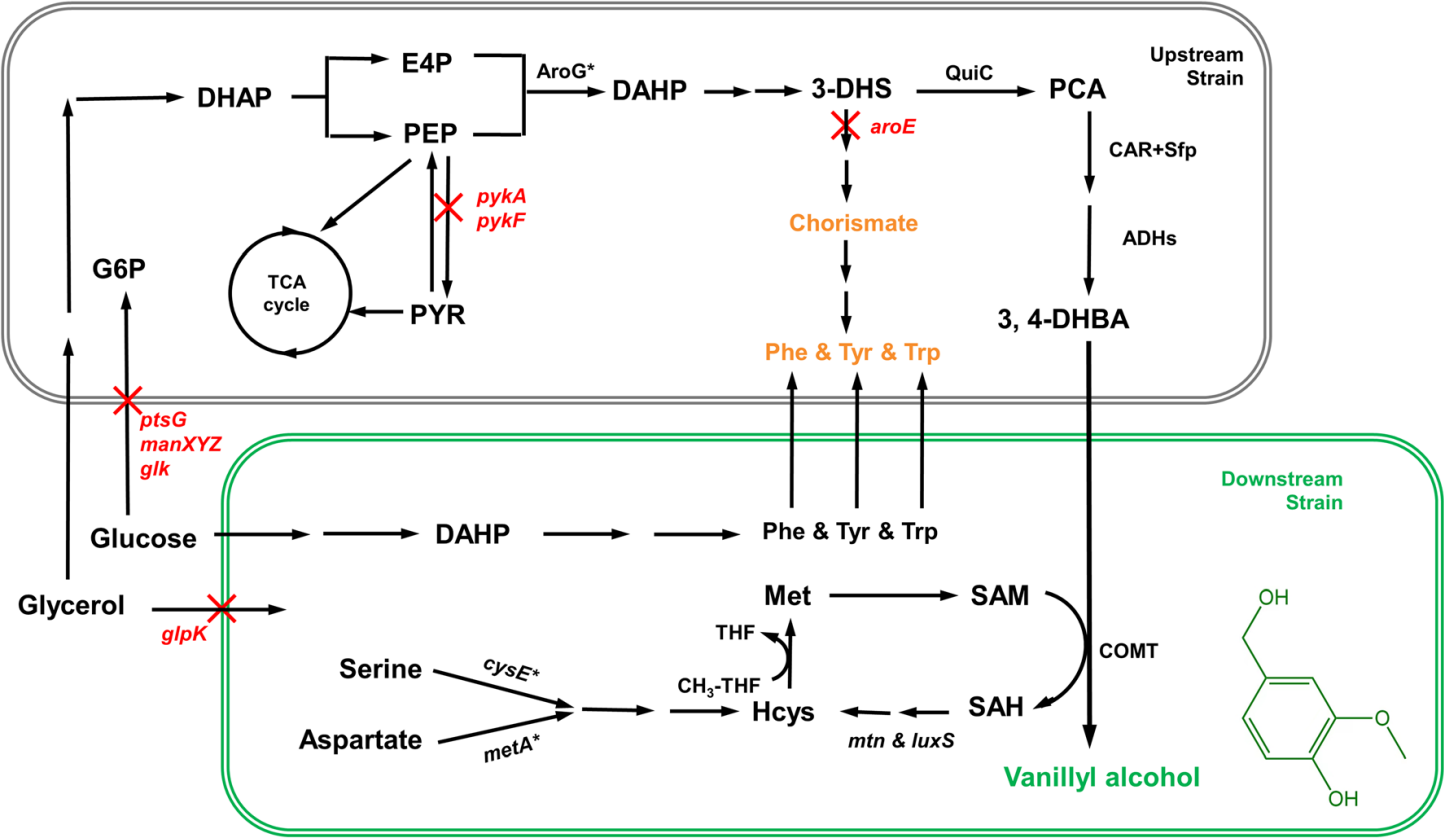
Design of *E. coli* coculture for vanillyl alcohol biosynthesis. *PEP* phosphoenolpyruvate, *E4P* erythrose 4-phosphate, *DAHP* 3-deoxy-D-arabinoheptulosonate 7-phosphate, *3-DHS* 3-dehydroshikimic acid, *SA* shikimate, *PCA* protocatechuic acid, *3,4-DHBA* 3, 4-dihydroxybenzyl alcohol, *SAM* S-adenosylmethionine, *Met* methionine, *Hcys* homocysteine, *SAH* S-adenosylhomocysteine, *QuiC* quinate dehydroshikimate dehydratase, *Car* carboxylic acid reductase, *Sfp* phosphopantetheinyl transferase, *ADHs* endogenous alcohol dehydrogenases, *COMT* caffeate O-methyltransferase, *LuxS* S-ribosylhomocysteine lyase, *Mtn* 5'-methylthioadenosine/S-adenosylhomocysteine nucleosidase, *glpK* encodes glycerol kinase, *glK* encodes glucokinase, *manXYZ* encodes mannose permease, *ptsG* encodes PTS glucose transport protein, *pykA/F* encode pyruvate kinases, *aroE* encodes shikimate dehydrogenase

In this study, we optimized vanillyl alcohol production in the monoculture by balancing expression levels of different modules and introducing the SAM regeneration cycle. However, the titer was still low with the accumulation of large amount of the intermediates. To solve this problem, *E. coli* cocultures were designed (Fig. 1), and the optimized system produced (559.4 ± 14.7) mg/L and (3890.2 ± 87.9) mg/L of vanillyl alcohol in shake flasks and bioreactor, respectively, which are the highest reported so far. This work demonstrates the potential of coculture engineering for efficient production of vanillin and its derivatives.

## Materials and methods

### Strains and plasmids

Strains and plasmids used in this study are listed in Table 1. *E. coli* strain DH5α was used as the host strain for plasmid construction. *E. coli* strain BW25113 and its derivatives were used for the feeding experiments and de novo biosynthesis. Plasmids pZE12-luc (high copy number), pCS27 (medium copy number), and pSA74 (low copy number) were used for constructing recombinant plasmids by enzyme digestion and ligation (Chen et al. 2017). Gene knockout was carried out using λ-RED recombination following the standard protocols (Datsenko and Wanner 2000).

**Table 1:** Strains and plasmids used in this study

Strains and plasmids	Description	Source
Strains		
BW25113	rrnBT14 ΔlacZWJ16 hsdR514 ΔaraBADAH33 ΔrhaBADLD78	Coli genome stock center
Lab01	BW25113, Δ*pykA*/*F*	(Li et al. 2019a)
Lab02	Lab01, Δ*ptsG*Δ*manXYZ*Δ*glk*	(Li et al. 2022)
Lab03	BW25113, Δ*glpk*	(Li et al. 2022)
YMC01	Lab01, pCS-CS, pZE-AQ	This study
YMC02	Lab01, pCS-CS, pZE-AUP	This study
YMC03	BW25113, pZE-COMT, pCS27	This study
YMC04	BW25113, pZE-COMT, pCS-LM	This study
YMC05	BW25113, pZE-COMT, pCS-MC	This study
YMC06	BW25113, pZE-COMT, pCS-LM-MC	This study
YMC07	Lab02, pZE-AQ-C, pCS-CS-LM	This study
YMC08	Lab02, pCS-AQ-LM, pZE-CS-C0.5	This study
YMC09	Lab02, pCS-AQ-LM, pZE-CS-C2.0	This study
YMC10	Lab02, pSA-AQ, pCS-LM, pZE-CS-C0.5	This study
YMC11	Lab02, pSA-AQ, pCS-LM, pZE-CS-C2.0	This study
YMC12	Lab02, pCS-CS, pZE-AQ	This study
YMC13	Lab03, pZE-COMT, pCS-LM	This study
YMC14	YMC12, Δ*aroE*	This study
Plasmids		
pZE12-luc	P_L_LacO1, *colE ori*, *luc*, *Amp*^r^, high copy	(Chen et al. 2017)
pCS27	P_L_LacO1, P15A ori, *Kan*^r^, medium copy	(Chen et al. 2017)
pSA74	P_L_LacO1, pSC101 *ori*, *Cm*^r^, low copy	(Chen et al. 2017)
pZE-COMT	pZE12-*luc*, *Arabidopsis thaliana* COMT encoding gene	(Chen et al. 2017)
pCS-CS	pCS27, *Mycobacterium marinum car* and *Bacillus subtilis sfp*	(Chen et al. 2017)
pZE-AQ	pZE12-*luc*, E. coli aroG^*^ and *Pseudomonas putida quiC*	This study
pZE-AUP	pZE12-*luc*, E. coli aroG^*^ and *ubiC, Pseudomonas aeruginosa pobA*	This study
pCS-LM	pCS27, *E. coli luxS* and *mtn*	This study
pCS-MC	pCS27, P_L_lacO1-metA^*^-cysE^*^	This study
pCS-LM-MC	pCS27, P_L_lacO1-*luxS-mtn* and P_L_lacO1-metA^*^-cysE^*^	This study
pZE- AQ-C	pZE12-*luc*, P_L_lacO1-aroG^*^-quiC and P_L_lacO1-COMT	This study
pCS-CS-LM	pCS27, P_L_lacO1-*car-sfp* and P_L_lacO1-*luxS-mtn*	This study
pSA-AQ	pSA74, P_L_lacO1-aroG^*^-*quiC*	This study
pCS-AQ-LM	pCS27, P_L_lacO1-aroG^*^-quiC and P_L_LacO1-*luxS-mtn*	This study
pZE-CSC0.5	pZE12-luc, Ptac-*car-sfp* and Plpp_0.5_-*COMT*	This study
pZE-CSC2.0	pZE12-luc, Ptac-*car-sfp* and Plpp_2.0_-*COMT*	This study

*Indicates feedback-resistant mutation.

### Cultivation conditions

LB medium containing 10 g tryptone, 5 g yeast extract and 10 g NaCl per liter was used for seed culture preparation. The M9 medium containing 15 g carbon source (glucose and/or glycerol), 6.78 g Na_2_HPO_4_, 0.5 g NaCl, 3 g KH_2_PO_4_, 1 g NH_4_Cl, 1 mM MgSO_4_, 0.1 mM CaCl_2_ and 2 g yeast extract per liter was used for shake flask experiments.

In shake flask experiments, single fresh colonies were picked into 4 mL LB medium and incubated at 37 °C, and 1 mL of overnight seed culture was transferred into 50 mL M9 medium and cultured at 30 °C and 200 rpm for 2 h. Then, protein expression was induced with 0.5 mM isopropyl-β-d-thiogalactopyranoside (IPTG). Feeding experiments were carried out to compare the efficiency of the different SAM-enhancing strategies. For this, the cell cultures of strains YMC04, YMC05 and YMC06 were induced with IPTG, supplemented with 1 g/L 3,4-DHBA at 2 h after inoculation, and continued to be cultured at 30 °C and 200 rpm. When necessary, ampicillin, kanamycin and chloramphenicol were added to the media at final concentrations of 100, 50 and 34 mg/mL, respectively.

In bioreactor experiments, the initial medium contained 15 g glucose, 5 g glycerol, 4 g (NH_4_)_2_SO_4_, 1.5 g KH_2_PO_4_, 3 g Na_2_HPO_4_, 4 g yeast extract and 30 mg/L VB_1_ per liter. The feeding solution contained 500 g/L glucose and 500 g/L glycerol. At the beginning, the seed cultures of strain YMC13 and YMC14 (50 mL each) were inoculated into the 3 L bioreactors (1 L working volume), and cultured at 30 °C. While the cell density at 600 nm (OD_600_) reached about 10, 0.5 mM IPTG was added to induce protein expression. During the whole culture process, the concentration of glucose was controlled under 5 g/L, the dissolved oxygen (DO) was controlled at 30% from 0 to 24 h and 8% after 24 h, and the pH was kept at 7.0.

For both the shake flask and the bioreactor experiments, samples were taken at regular time intervals for analysis of cell growth and production. Cell growth was monitored by measuring the OD_600_. The concentrations 3,4-DHBA, protocatechuic acid (PCA), and vanillyl alcohol were analyzed by HPLC.

### HPLC analysis

The standard of (3,4-DHBA), protocatechuic acid (PCA), and vanillyl alcohol were purchased from Macklin (Shanghai Macklin Biochemical Co., Ltd). HPLC (HITACHI) equipped with a reverse-phase Diamonsil C_18_ column (Diamonsil 5 μm, 250 mm × 4.6 mm) and an UV–vis detector was used for analysis of PCA, 3,4-DHBA and vanillyl alcohol. The mobile phase contains solvent A (water with 0.1% formic acid) and solvent B (100% methanol) with a flow rate of 0.8 mL/min. The column temperature was set at 35 °C. The following gradient was set: 5% to 50% solvent B for 20 min, 100% solvent B for 2 min, 100% to 5% solvent B for 2 min and 5% solvent B for an additional 5 min. Quantification was based on the peak areas at specific wavelengths (254 nm for PCA, 280 nm for 3,4-DHBA and vanillyl alcohol).

## Results and discussion

### Comparision of 3,4-DHBA biosynthesis via the 3-DHS and the 4-HBA pathways

**Fig. 2:**
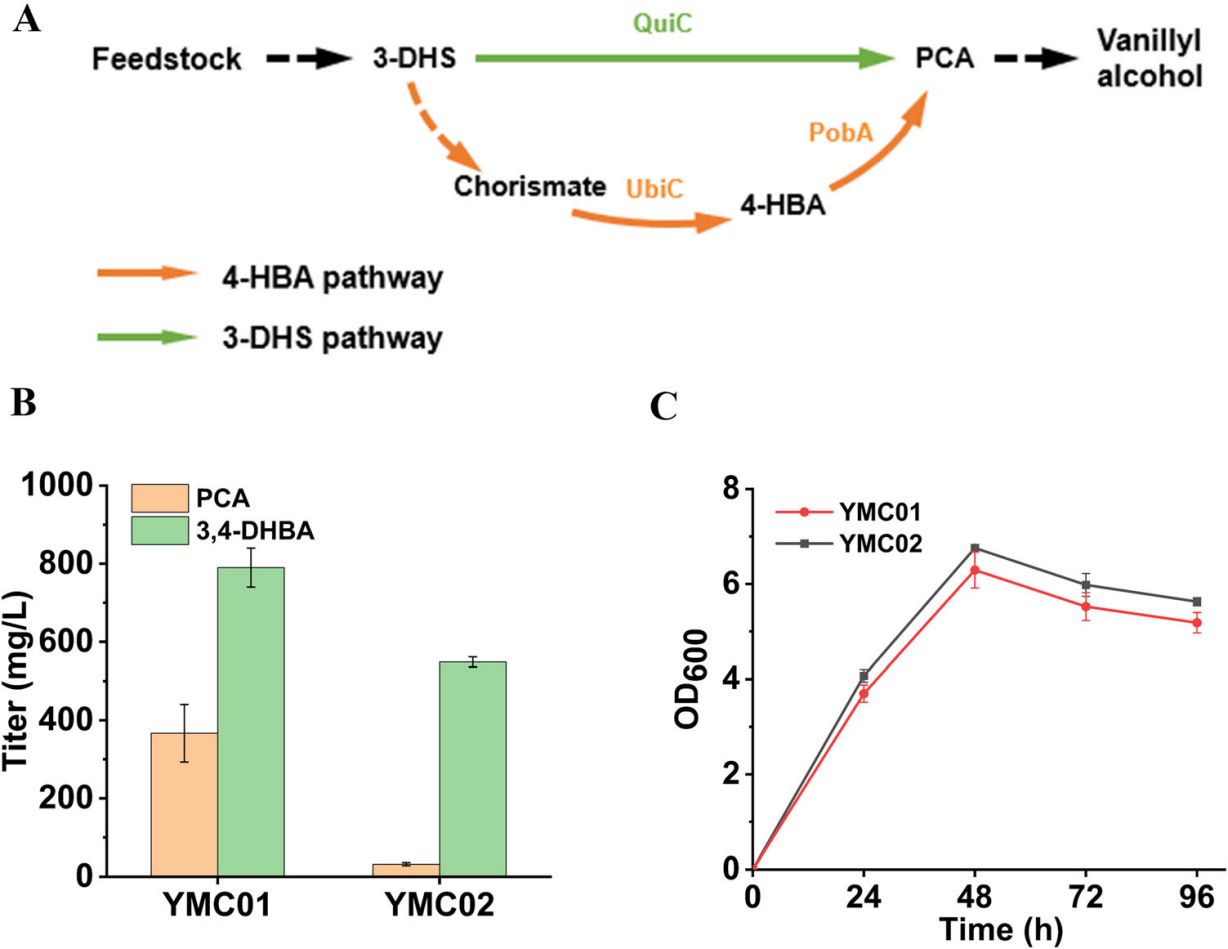
Comparison of the 3-DHS pathway (YMC01) and the 4-HBA pathway (YMC02) for 3,4-DHBA production. **A** Schematic of the two pathways, **B** The titers of PCA and 3, 4-DHBA by YMC01 and YMC02 at 96 h, **C** Growth profiles of strains YMC01 and YMC02

Vanillyl alcohol can be produced either via the 3-DHS pathway or the 4-HBA pathway (Fig. 2A). As the last step catalyzed by COMT is rate-limitng, we first compared the efficiency of the two pathways by producing the penultimate compound 3, 4-DHBA. For the 3-DHS pathway, plasmid pZE-AQ expressing *E. coli* feedback-resistant aroG^*^ and *P. putida* 3-DHS dehydratase gene *quiC* was constructed. AroG^*^ catalyzes the committed step of the shikimate pathway while QuiC converts 3-DHS to PCA. The previously constructed plasmid pCS-CS produces CAR and its activator SFP, catalyzing reduction of PCA to the aldehyde. The aldehyde can be reduced to 3,4-DHBA by endogenous reductases. Plasmids pZE-AQ and pCS-CS were co-transfered into strain BW25113Δ*pykA*/*F*, generating strain YMC01. Strain BW25113Δ*pykA*/*F* was used instead of the wild-type BW25113. Genes *pykA* and *pykF* encode pyruvate kinases which convert PEP to pyruvate. Deletion of *pykA*/*F* can conserve PEP and increase the flux to the shikimate pathway. In shake flask experiment with glycerol as the carbon source, strain YMC01 produced (790.0 ± 49.9) mg/L 3, 4-DHBA and (366.7 ± 73.7) mg/L PCA (Fig. 2B). Similarly, for the 4-HBA pathway, plasmid pZE-AUP was constructed, expressing E. coli aroG^*^ and *ubiC*, and *P. aeruginosa pobA*. Plasmids pZE-AUP and pCS-CS were co-transfered into strain BW25113Δ*pykA*Δ*pykF*, generating strain YMC02. Strain YMC02 produced (548.8 ± 12.9) mg/L 3,4-DHBA and (32.0 ± 4.1) mg/L PCA at 96 h (Fig. 2B). The two strains showed similar growth profiles, and cell densities reached maximum at 48 h (Fig. 2C). The results showed that the 3-DHS pathway has higher efficiency than the 4-HBA pathway. Thus, the former was used in the following study for vanillyl alcohol production.

### Improving 3,4-DHBA methylation by strengthening SAM regeneration cycle

After optimizing 3,4-DHBA production from glycerol, we next tested the conversion capability of COMT by feeding experiment. The wild-type strain BW25113 was co-transformed with plasmids pZE-COMT and pCS27 (empty vector), generating strain YMC03. When fed with 1 g/L 3,4-DHBA, strain YMC03 produced (305.7 ± 8.2) mg/L vanillyl alcohol at 16 h. SAM is a co-substrate of COMT, and its supply is essential to the conversion efficiency. In a previous study of vanilate biosynthesis, two strategies have been adopted (Kunjapur et al. 2016). One is to strengthen SAM regeneration by expressing *luxS* and *mtn* (LM module) and the other is to enhance its biosynthesis by expressing two key genes metA* and cysE* (MC module).

**Fig. 3:**
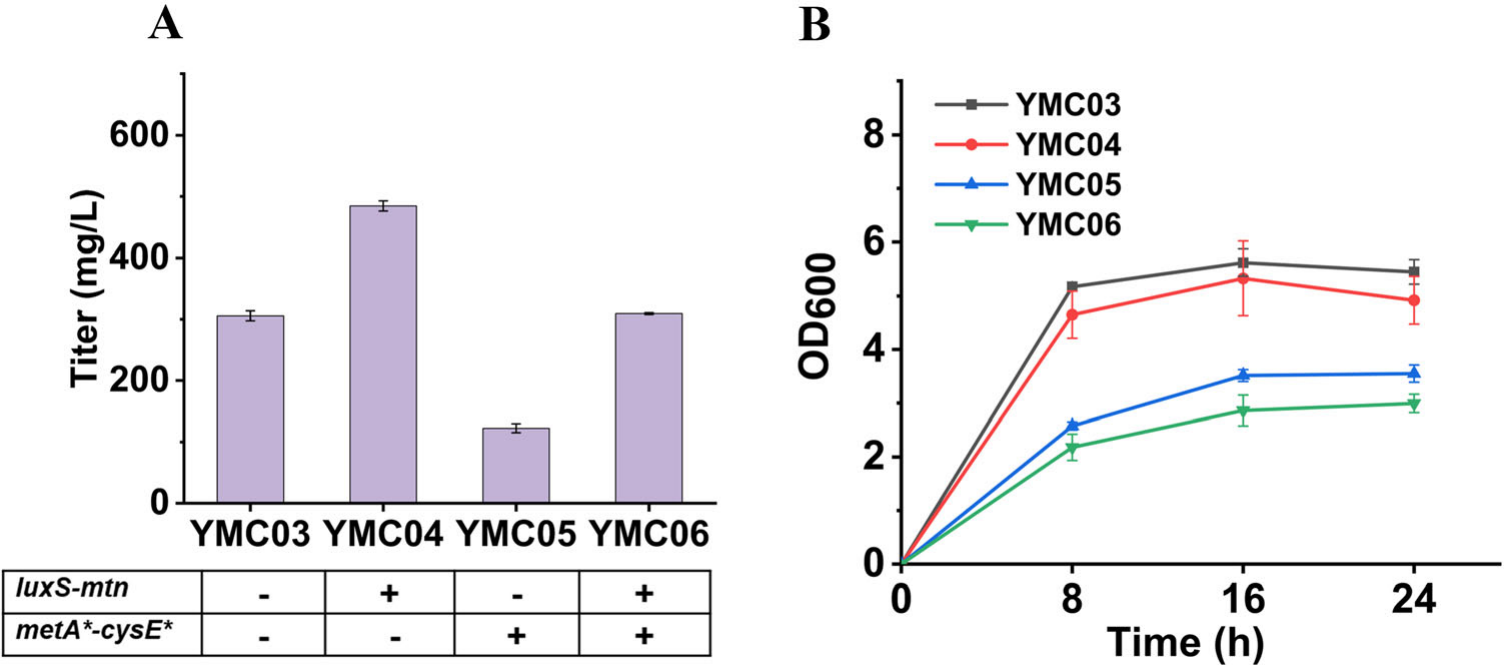
Effects of strengthening S-adenosylmethionine regeneration or/and biosynthesis on the methylation capacity of COMT. **A** Vanillyl alcohol titers of different strains at 16 h, **B** Growth profiles of the four strains. 3,4-DHBA (1 g/L) was fed to the cell cultures. See Table 1 for the genetic information of the strains

To test the effectiveness of these two strategies, plasmids pCS-LM, pCS-MC and pCS-LM-MC were constructed, and co-transferred with plasmid pZE-COMT into BW25113, respectively, generating strains YMC04, YMC05 and YMC06. In the feeding experiment, strain YMC04 produced 484.6 ± 8.3 mg/L vanillyl alcohol at 16 h, which is 58.5% higher than that of YMC03. However, strain YMC05 produced significantly less vanillyl alcohol (122.5 ± 7.3 mg/L). Further introducing LM module (strain YMC06) recovered vanillyl alcohol to 309.5 ± 1.4 mg/L (Fig. 3A). As shown in Fig. 3B, overexpressing metA* and cysE* drastically reduced cell growth of strains YMC05 and YMC06, which may be attributed to the accumulation of toxic S-adenosylhomocysteine (SAH) (Roe et al. 2002). The results showed that enhancement of SAM regeneration rather than SAM biosynthesis is an effective way to increase methylation efficiency, and the ineffectiveness of the latter strategy is mainly due to its negative effect on cell growth.

### Optimizing vanillyl alcohol biosynthesis in monoculture

**Fig. 4:**
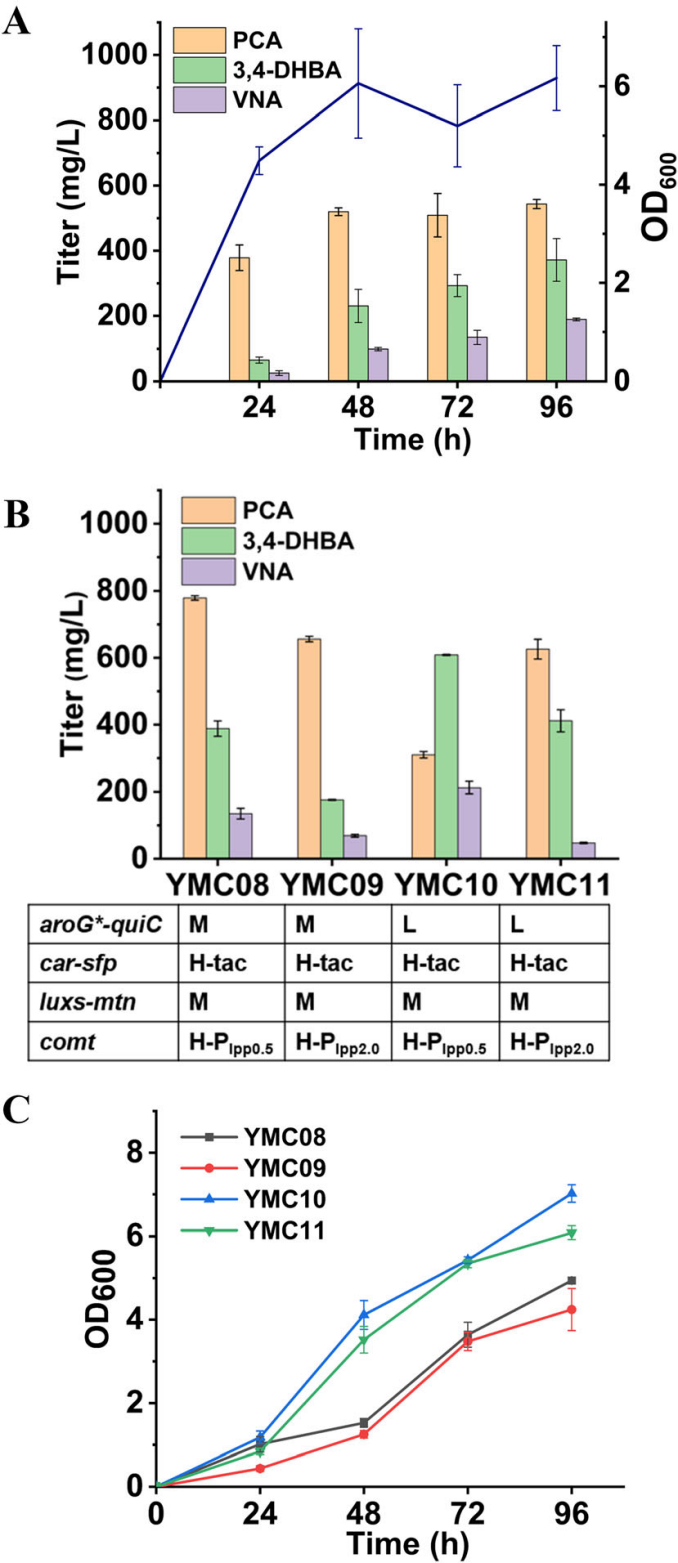
De novo biosynthesis of vanillyl alcohol in monocultures. **A** Vanillyl alcohol production by strain YMC07, **B** Modular optimization of gene expression for vanillyl alcohol, **C** Growth profiles of strains YMC08, YMC09, YMC10 and YMC11. See Table 1 for the genetic information of the strains. *H* high copy, *M* medium copy, *L* low copy. Data shown are mean ± SD (*n* = 3 biological replicates)

To achieve de novo biosynthesis of vanillyl alcohol, we assembled the full pathway in one strain. For this purpose, plasmids pZE-AQ-C and pCS-CS-LM were constructed, and co-transferred into strain Lab02, producing strain YMC07. Notably, Lab02 is a derived strain of Lab01 and is unable to use glucose as the carbon source (Li et al. 2022). In shake flask experiment with glycerol as the carbon source, strain YMC07 produced (189.0 ± 4.2) mg/L vanillyl alcohol with the accumulation of (543.4 ± 14.1) mg/L PCA and (371.7 ± 35.6) mg/L 3,4-DHBA (Fig. 4A). The accumulation of large amount of the intermediates indicates that the efficiency of the last two enzymatic steps is insufficient. To solve this problem, modular optimization was conducted by adjusting plasmid copy number and promoter strength. As PCA was accumulated in large amounts, the AQ module was thus moved from the high-copy (H) to the medium (M) or the low-copy (L) plasmids. Similarly, the CS and C modules were moved to the high-copy plasmid, and two strong constitutive promoters (Plpp0.5 and Plpp2.0) were used to control expression of COMT gene. As a result, four strains (YMC08, YMC09, YMC10 and YMC11) were produced. However, modular optimization only led to modest improvement in vanillyl alcohol production, with the best titer of (212.6 ± 18.4) mg/L by strain YMC10 (Fig. 4B, C).

Production of 1 mol 3,4-DHBA from PCA consumes 2 mol NADPH and 1 mol ATP. Moreover, methylation reactions are among the most energy-consuming processes in nature. The regeneration of one active methyl group in the form of SAM costs 12 ATP equivalents (Nyyssola and Leisola 2001). Thus, it seems difficult to balance gene expression and meet the demand for energy and redox power in a single cell.

### Optimizing vanillyl alcohol biosynthesis in *E. coli* coculture

Since optimization of vanillyl alcohol biosynthesis in a single cell was not successful, we turned to explore the coculture strategy. In the design, two BW25113 derived strains Lab02 (BW25113Δ*ptsG*Δ*manXYZ*Δ*glk*) and Lab03 (BW25113Δ*glpK*) were used as the upstream and the downstream hosts, respectively (Li et al. 2022). When glucose and glycerol are use as the mixed carbon source, the two strains will use glycerol and glucose, respectively, which is supposed to reduce the competition between them. Lab02 was transformed with plasmids pCS-CS, pZE-AQ, producing strain YMC12 responsible for 3,4-DHBA production from glycerol. Lab03 was transformed with plasmids pZE-COMT and pCS-LM, producing strain YMC13 responsible for conversion of 3,4-DHBA to vanillyl alcohol.

**Fig. 5:**
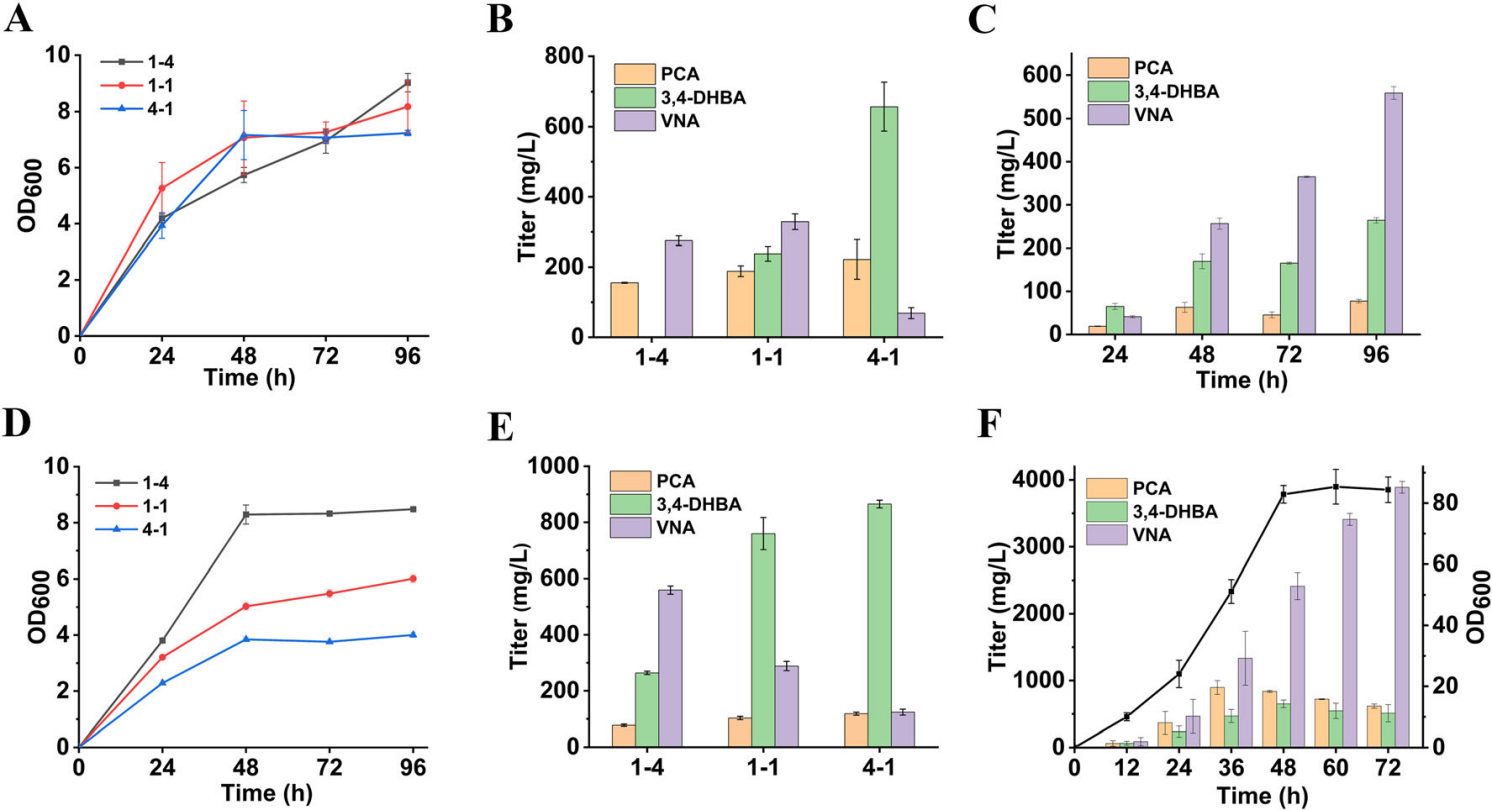
Vanillyl alcohol biosynthesis by *E. coli* cocultures. **A** Growth profiles of the YMC12/YMC13 coculture with different inoculation ratios, **B** Vanillyl alcohol titers by the YMC12/YMC13 coculture at 96 h with different inoculation ratios, **C** Growth profiles of the YMC14/YMC13 coculture with different inoculation ratios, **D** Vanillyl alcohol titers by the YMC14/YMC13 coculture at 96 h with different inoculation ratios, **E** Accumulation curves of VNA and the intermediates by the YMC14/YMC13 coculture, **F** Scale-up production of vanillyl alcohol in bioreactors with the inoculation ratio of 1:4. *VNA* vanillyl alcohol. Data shown are mean ± SD (*n* = 3 biological replicates)

In shake flask coculture experiment, strains YMC12 and YMC13 were inoculated at three initial ratios (1:4, 1:1 and 4:1). The growth profiles were roughly comparable at the three ratios (Fig. 5A). The inoculation ratio showed significant effect on vanillyl alcohol production and intermediate accumulation (Fig. 5B). Generally, the accumulation of the intermediates (PCA and 3,4-DHBA) increased with the increasing ratio of the upstream strain. The best vanillyl alcohol titer (328.9 ± 22.3 mg/L) was achieved at inoculation of 1:1, which is 54% higher than that in the YMC10 monoculture. Meanwhile, (188.3 ± 15.2) mg/L PCA and (238.0 ± 20.8) mg/L 3,4-DHBA were accumulated (Fig. 5B).

To further improve the performance of the coculture system, we chose to knock out *aroE* gene in the upstream strain, producing strain YMC14. Knockout of *aroE* can, on one hand, block the synthesis of aromatic amino acids and other essential metabolites such as 4-hydrxybezoate and 4-aminobezoate, and on the other hand, conserve and direct more carbon flux to the target pathway. Thus, it is expected to reduce cell growth but improve production capacity of the upstream strain, making room for the rating-limiting downstream strain in the coculture.

In the YMC14/YMC13 coculture, the holistic growth profiles were negatively correlated with the inoculation ratio of the upstream strain. The OD_600_ value reached 8.48 ± 0.02 at the ratio of 1:4, but just reached 4.01 ± 0.09 at the ratio of 4:1 (Fig. 5C). Interestingly, vanillyl alcohol titer was also negatively correlated with the inoculation ratio of the upstream strain. At the ratio of 1:4, the best titer reached (559.4 ± 14.7) mg/L at 96 h, which is 70% higher than that of the YMC12/YMC13 coculture (Fig. 5D). Meanwhile, (77.4 ± 4.2) mg/L PCA and (264.0 ± 6.2) mg/L 3, 4-DHBA were still accumulated. The results indicate that due to *aroE* knockout strain YMC14 has improved carbon flux through the shikimate pathway than strain YMC12. At the low inoculation ratio (1:4), the upstream strain can still provide sufficient precursors. Vanillyl alcohol titer could potentially be improved by further lowering the upstream ratio. The production curve showed that the vanillyl alcohol titer kept increasing during the cultivation time (Fig. 5E).

To explore the scale-up potential, feedback fermentation of the YMC14/YMC13 coculture was performed in 3 L bioreactors containing 1 L initial fermentation broth. The inoculation was also kept at 1:4. As the show in Fig. 5F, the cells grew fast within the first 48 h and the OD_600_ value reached 82.9 ± 2.9 at 48 h. Thereafter, the cell density remained stable. Vanillyl alcohol titer kept increasing during the cultivation period and the titer reached (3890.2 ± 87.9) mg/L at 72 h, with the accumulation of (617.0 ± 31.3) mg/L PCA and (510.5 ± 128.8) mg/L 3, 4-DHBA. According to the results, the methyltransferase is an obvious bottleneck in vanillyl alcohol biosynthesis. To further improve the production efficiency, in the future this problem should be tackled by strategies such as enzyme mining and protein engineering.

## Conclusion

In this study, we first aimed to achieve efficient vanillyl alcohol production in a single cell. However, stepwise optimization of the pathway, including pathway selection, enhancement of SAM regeneration and modular optimization led to no significant improvement in the production. The two precursors were still accumulated in large amounts. This suggests that it may be challenging and laborious to balance the pathway and the demands of enzymes for energy and cofactors. Thus, by applying the coculture engineering strategy, the pathway was divided and distributed into two *E. coli* cells. This effort led to the highest vanillyl alcohol titer reported so far, demonstrating the potential of coculturing engineering in biosynthesis of valuable compounds.

## Data Availability

All data generated or analyzed during this study are included in this published article.
